# Re-Union of the Alumni of the Jefferson Medical College

**Published:** 1872-01-01

**Authors:** Jewing Mears, R. J. Dunglison

**Affiliations:** 222 S. Sixteenth St.; 636 No. Eighteenth St.


### RE-UNION OF THE ALUMNI OF THE JEFFERSON MEDICAL COLLEGE.

The Alumni Association of the Jefferson Medical College proposes to hold a social re-union during the meeting of the American Medical Association in Philadelphia in May next. The Alumni of the College are cordially invited to attend. Those who expect to be present are requested to send their names and addresses to either of the undersigned secretaries.

J. EWING MEARS, M. D.,

222 S. Sixteenth St.

R. J. DUNGLISON,

636 No. Eighteenth St.

